# Mass Spectrometry–Based Proteomics of Epithelial Ovarian Cancers: A Clinical Perspective

**DOI:** 10.1016/j.mcpro.2023.100578

**Published:** 2023-05-19

**Authors:** Liujia Qian, Rui Sun, Zhangzhi Xue, Tiannan Guo

**Affiliations:** 1 iMarker Lab, Westlake Laboratory of Life Sciences and Biomedicine, Key Laboratory of Structural Biology of Zhejiang Province, School of Life Sciences, Westlake University, Hangzhou, Zhejiang, China; 2 Institute of Basic Medical Sciences, Westlake Institute for Advanced Study, Hangzhou, Zhejiang, China; 3 Research Center for Industries of the Future, Westlake University, Hangzhou, Zhejiang, China

**Keywords:** epithelial ovarian cancer, proteomics, early diagnosis, therapeutic targets, clinical trials

## Abstract

Increasing proteomic studies focused on epithelial ovarian cancer (EOC) have attempted to identify early disease biomarkers, establish molecular stratification, and discover novel druggable targets. Here we review these recent studies from a clinical perspective. Multiple blood proteins have been used clinically as diagnostic markers. The ROMA test integrates CA125 and HE4, while the OVA1 and OVA2 tests analyze multiple proteins identified by proteomics. Targeted proteomics has been widely used to identify and validate potential diagnostic biomarkers in EOCs, but none has yet been approved for clinical adoption. Discovery of proteomic characterization of bulk EOC tissue specimens has uncovered a large number of dysregulated proteins, proposed new stratification schemes, and revealed novel targets of therapeutic potential. A major hurdle facing clinical translation of these stratification schemes based on bulk proteomic profiling is intra-tumor heterogeneity, namely that single tumor specimens may harbor molecular features of multiple subtypes. We reviewed over 2500 interventional clinical trials of ovarian cancers since 1990 and cataloged 22 types of interventions adopted in these trials. Among 1418 clinical trials which have been completed or are not recruiting new patients, about 50% investigated chemotherapies. Thirty-seven clinical trials are at phase 3 or 4, of which 12 focus on PARP, 10 on VEGFR, 9 on conventional anti-cancer agents, and the remaining on sex hormones, MEK1/2, PD-L1, ERBB, and FRα. Although none of the foregoing therapeutic targets were discovered by proteomics, newer targets discovered by proteomics, including HSP90 and cancer/testis antigens, are being tested also in clinical trials. To accelerate the translation of proteomic findings to clinical practice, future studies need to be designed and executed to the stringent standards of practice-changing clinical trials. We anticipate that the rapidly evolving technology of spatial and single-cell proteomics will deconvolute the intra-tumor heterogeneity of EOCs, further facilitating their precise stratification and superior treatment outcomes.

## Highlights

• Systematic review of proteomics studies of EOCs from a clinical perspective.
• Systematic review of ∼2500 clinical trials of ovarian cancer since 1990.
• Advances in proteomics show promise in customized therapies for EOCs.

## Graphical Abstract

**Figure:**
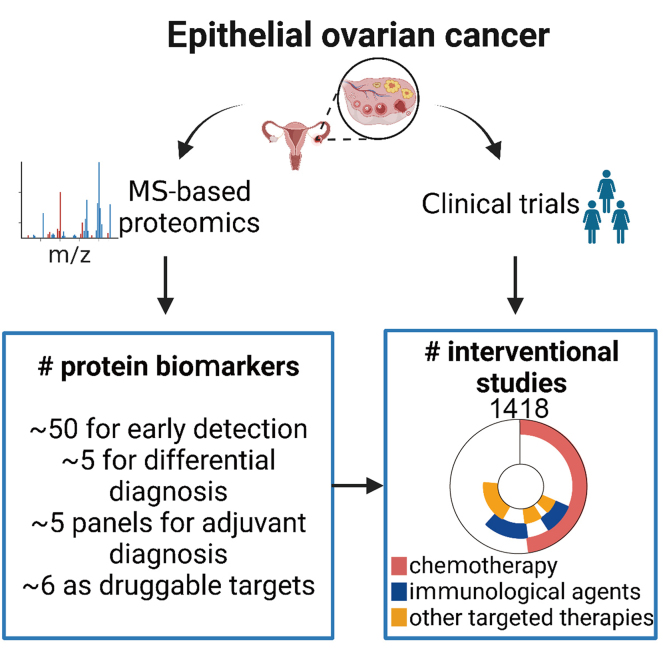

## In Brief

We systematically reviewed the MS-based proteomics studies of epithelial ovarian cancer, and over 2000 clinical trials since 1990. None of MS-based protein assays has been adopted in clinic, and none of the drug targets firstly discovered by proteomics has advanced to phase 3 or 4. Stringent standards for study design of proteomics are required, and more in-depth analyses of dysregulated proteins are essential.

## Clinical Challenges in Epithelial Ovarian Cancer

Ovarian cancer is the eighth most common cancer and one of the most fatal in females worldwide (1). Epithelial ovarian cancer (EOC) accounts for nearly 90% of all ovarian cancers. It is a heterogeneous disease composed of five histological subtypes: high-grade serous ovarian cancer (HGSOC), low-grade serous ovarian carcinoma (LGSOC), mucinous carcinoma, endometrioid carcinoma, and clear cell carcinoma. HGSOC accounts for approximately 75% of all EOCs. EOC subtypes have different progenitors, molecular alterations, and clinical features.

The 5-year relative survival rate of ovarian cancer is about 50% (2). Multiple factors contribute to the high mortality (3). First, as early-stage ovarian cancer is difficult to detect, about 75% of patients are diagnosed at an advanced stage. The 5-year relative survival of such patients is significantly lower (30%) compared to early-stage patients (93%) (2). Second, although different histological subtypes with distinct genomic characteristics are known, standard first-line treatments including chemotherapy, targeted therapy and immunotherapy have been developed and customized mainly for HGSOC but are also being applied to other relatively rare histological subtypes although the molecular rewiring of these tumors remains unclear. Third, tumors of the same histological subtype are known to exhibit different responses to therapy, raising the need for a higher resolution molecular and functional cellular taxonomy of ovarian cancers. Fourth, there is no effective treatment against relapse and acquired resistance to chemotherapy. Although 80% of patients respond well to the initial platinum-based chemotherapy, most relapse after a short interval, and some even develop acquired resistance (4). Some targeted drugs, such as poly(ADP-ribose) polymerase inhibitors (PARPi) (5, 6) and VEGF inhibitors including bevacizumab (7, 8, 9), have been approved and used in clinical practice for their benefits against recurrence. However, bevacizumab combined with chemotherapy has shown no significant improvement in overall survival (OS) in either platinum-sensitive (7, 8) or platinum-resistant (9) cohorts. In addition, reversions of germline *BRCA1/2* mutations have been reported in platinum-resistant patients (10, 11, 12, 13), indicating development of PARPi resistance (10, 11, 13). Thus, deep molecular characterization of recurrent and treatment-resistant ovarian cancers is essential for developing new effective treatments.

## Histological and Molecular Stratification of EOC

Shih *et al.* (14) proposed a dualistic model of EOC based on morphological and molecular genetic analyses in 2004. Type I EOC refers to the tumors which develop stepwise from borderline tumors with relatively low proliferative activity, while type II EOC is more aggressive. According to this model, HGSOCs are type II tumors, whereas the remaining four EOC histological subtypes are type I tumors.

HGSOC is the most molecularly characterized EOC. Multiple genetic molecular changes have been reported in HGSOC tumors. Nearly all (96%) contain *TP53* mutations that lead to substantial chromosomal structural variations (15). Also common are point mutations in oncogenes and tumor suppressor genes, such as *BRCA1* (12%), *BRCA2* (11%), and *NF1* (4%) (16). Of note, both *BRCA1* and *BRCA2* are involved in the homologous recombination (HR) pathway that modulates DNA repair. Interestingly, about 50% of HGSOC tumors harbor genetic or epigenetic aberrancy in the HR pathway (16), resulting in defective HR-dependent DNA repair and favorable responses to platinum-based therapy (10). The most common focal copy number alteration in HGSOC is the amplification of *CCNE1*, a cell cycle regulator, occurring in nearly 30% of patients without HR deficiency (16). *CCNE1* amplification is associated with unfavorable prognosis (10, 17).

HGSOC may be further divided into four subtypes based on mRNA expression (16, 18, 19). In 2008, the Australian Ovarian Cancer Study classified about 100 HGSOC tumors into four groups based on differences in tumor microenvironments inferred from microarray data (16, 18). Among these, tumors with heightened stromal response conferred the worst prognosis while patients whose tumors exhibited low stromal response and high immune signature survived longer. The Cancer Genome Atlas (TCGA) also reported four molecular subtypes of 489 HGSOC tumors based on the expression of ∼1500 genes, although no prognostic differences were observed (log-rank test, *p* value = 0.26) (16). However, Konecny *et al.* (19) applied the TCGA classifier to a cohort of 174 HGSOC patients from Mayo Clinic, and observed prognostic significance (log-rank test, *p* value = 0.004) among the four subtypes. In addition, *de novo* subtyping of the Mayo Clinic cohort yielded four clusters with similar functional signatures as TCGA (log-rank test, *p* value = 0.006). Of note, a single HGSOC tumor may display molecular characteristic of multiple subtypes. For this reason, 42% to 82% of tumors from the TCGA and Mayo Clinic cohorts were assigned to at least two subtypes (16, 19, 20). The clinical validity of these classifiers remains to be further justified in independent cohorts.

Type I EOC tumors harbor molecular features distinct from HGSOC. LGSOC is frequently characterized by mutations in *KRAS* (40%) and *BRAF* (12%) (21). *ARID1A* mutations, common in both clear cell EOC (46%) and endometrioid carcinoma (30%), are associated with the malignant transformation of endometriosis (22, 23). In addition, frequent mutations of *PIK3CA* (33%) (24) and overexpression of the IL6-STAT3-HIF pathway (25) have been observed in clear cell EOC. Finally, mucinous carcinoma is the least common type of EOC and is more likely to be diagnosed at early stages. Compared with the other histological subtypes, mucinous carcinomas harbor more frequent *KRAS* mutations (45%) (26).

## Therapies Against EOCs

For more than 20 years, widely adopted primary treatment of EOCs consists of debulking surgery followed by first-line chemotherapy with carboplatin and paclitaxel (27). Several trials have tested the efficacy of combining anti-angiogenic drugs with chemotherapies. Bevacizumab, a therapeutic antibody against VEGF, in combination with chemotherapy has been reported to prolong the progression-free survival (PFS) of patients with EOC, especially those with platinum-resistant tumors (9, 28, 29). This combination therapy has been approved by the FDA for patients with advanced-stage EOC after initial surgical resection. Olaparib (AZD2281), a PARP inhibitor inactivating DNA repair, is an effective therapy for EOCs with defective HR (30, 31) and has been approved as maintenance treatment against *BRCA1/2*-mutated, advanced ovarian cancer (5, 6).

Besides the vasculature surrounding tumor cells, tumor-infiltrating CD8+ T cells (32, 33, 34) and CD20+ B cells (32) are associated with delayed recurrence and improved survival, while the presence of immunosuppressive cells, such as Treg cells (34, 35, 36) and B7-H4 macrophages (35), are associated with unfavorable prognoses. Thus, some trials have investigated the benefits of single-agent immunotherapy or chemoimmunotherapy, such as immune checkpoint blockade (37, 38, 39, 40), immunomodulatory drugs (41, 42), and adoptive cell therapy (43), in patients with EOC, especially those with advanced stage or recurrent platinum-resistant tumors. However, most of these monotherapies have yielded disappointing results, probably because of relatively low mutational burdens in EOCs (44). Trials have tested combining immunotherapy with chemotherapy (39) or anti-angiogenic therapy (45). The phase III JAVELIN Ovarian 200 clinical trial reported that combining avelumab, a PD-L1 inhibitor, with pegylated liposomal doxorubicin did not significantly improve prognosis in all patients, but only in a subgroup of patients with positive expression of PD-L1 and CD8 (39). A phase II clinical trial evaluated the clinical activity of combining the PD-L1 inhibitor Nivolumab with Bevacizumab, and found a high objective response rate (ORR, 40%) in platinum-sensitive patients (45). However, those with negative PD-L1 yielded higher ORR (45.5%) than those with positive PD-L1 (14.3%) (45). These contradictory findings suggest that the response to immunotherapy may be determined by multiple factors which remain to be fully understood. In summary, as only a few therapeutic targets have been verified in clinical trials, first-line therapy for EOC remains surgery and chemotherapy after more than 2 decades. More targets vulnerable or sensitive to new therapies are urgently required to improve clinical outcomes.

Neoadjuvant chemotherapy (NACT) for advanced stage disease has been reported to minimize residual disease after interval debulking surgery (46). However, multiple clinical trials have shown no impact of NACT on overall survival compared with primary debulking surgery (47, 48, 49, 50). Although a subset of patients with EOC benefit from NACT, it is currently not possible to identify such patients prospectively.

## Advances in Mass Spectrometry–Based Proteomics

Mass spectrometry (MS)–based proteomics with increased throughput (51, 52, 53, 54) offers unique tools to discover novel biomarkers and therapeutic targets for EOC. Data-dependent acquisition (DDA) and data-independent acquisition (DIA) are the two major MS strategies for unbiased biomarker discovery, currently enabling the characterization of over 10,000 proteins from minute amounts of clinical specimens. In DDA mode, precursor ions are selected based on signal intensity for fragmentation, and this semirandom peptide sampling strategy compromises the data reproducibility and leads to relatively high rates of missing values. Extensive fractionation of complex peptide mixture before LC-MS analysis could reduce the complexity of co-eluting peptide precursors, and improves the proteome coverage; however, this also compromises its throughput. Recent progresses in stable isotopic labeling technologies such as TMTpro (55) has substantially improved the throughput of DDA-MS without compromising the depth. As up to 16 or 18 samples can be analyzed simultaneously with TMTpro, this technique could be adopted to analyze up to several hundred specimens. When multiple batches of TMTpro experiments are needed, at least one channel has to be reserved as a control sample to align the quantitative results across batches (56). Comparative analysis of TMT data from different studies is challenging since usually they do not have a common control sample. While DIA mode overcomes the stochastic sampling problem by systematic and unbiased fragmentation of all ionized peptides in a predefined mass range, hence offering higher reproducibility, relatively low missing values, improved accuracy and sensitivity. DIA-MS is mostly used for unfractionated samples, therefore, it allows high-throughput proteomic analysis (53). DIA-MS can be readily applied to analyze up to several thousand specimens with a high degree of reproducibility and reasonable depth. The unique and major challenge for DIA-MS is the computational deconvolution of the mixed spectral data. Although a DIA map in principle contains spectral data for all ionized peptides, to identify and quantify them remains a technical challenge, and the software tools for interpreting DIA data continues to evolve (57).

When the biomarker candidates are narrowed down to tens to hundreds, targeted MS strategies, like multiple/selected reaction monitoring (M/SRM) and parallel reaction monitoring (PRM), are better suited to verify them in high throughput in large cohorts. M/SRM and PRM allow highly reproducible and sensitive measurements of selected proteins, and could be potentially applied in clinical practice.

In recent years, multiple approaches have been developed to interrogate spatial and/or cellular, in some cases subcellular, heterogeneity of cancers (58, 59). SCoPE-MS (single-cell proteomics by MS) introduces a booster channel of hundreds of cells into single-cell samples using multiplex TMT reagents to increase overall signals (58). Brunner *et al.* (59) developed single-cell proteomics based on diaPASEF which, when further coupled with miniaturization of sample preparation and low-flow chromatography, achieved a 10-fold increase in sensitivity. In addition, multiple spatial methods, such as antibody-based imaging, particle beam scans, and direct microscopy coupled with MS-based proteomics, help retrieve molecular details on the interactions between tumor cells and their microenvironment, the organization of immune cells, blood vessels, and fibroblasts in clinical specimens (60). Recently, ProteomEx (Expansion Proteomics) integrated tissue expansion with DIA-MS, offering an easily accessible approach for spatially resolved proteomics at ∼160 μm lateral resolution (61). Due to the nature of this review, we cannot catalog all the exciting MS-based proteomics methods here. Interested readers are referred to other recent reviews (52, 53, 60, 62).

Many previous reviews have summarized MS-based proteomics studies of ovarian cancers (63, 64, 65, 66, 67, 68, 69, 70, 71, 72, 73, 74, 75). Several focus on specific aspects. Deng *et al.* (67) focused on biomarkers of chemoresistance, while Li *et al.* (69) investigated mitochondrial proteomics. Advances in proteomic technologies and biomarker screening are the focus of Luu *et al.* (75). Kulasingam *et al.* (63) provided insights on biomarker discovery by integrating high-throughput omics technologies developed before 2010, highlighting the promise of MS-based proteomics. Xiao *et al.* (73) summarized biomarkers identified by multi-omics approaches. Other reviews aimed to provide comprehensive overviews of diagnostic biomarkers, molecular stratification, and resistance-associated biomarkers for ovarian cancers (64, 65, 66, 68, 70, 71, 72, 74), but none attempted to apply stringent clinical assessments on the likely impact of progress in ovarian cancer proteomics on clinical management and patient outcomes.

## Diagnostic Methods Driven by MS-Based Proteomics

New biomarkers for the detection of early-stage EOC could potentially reduce its mortality, however, it is still challenging. First, due to the relatively low incidence of EOC and the potential complications of invasive confirmatory laparoscopy, the specificity of a valid screening test should be as high as 99.6% (76). Second, most studies are based on clinically apparent ovarian cancer samples which are often late-stage cancer. However, analyzing samples from early-stage EOC is essential to develop a screening test with high sensitivity.

**Fig. 1:**
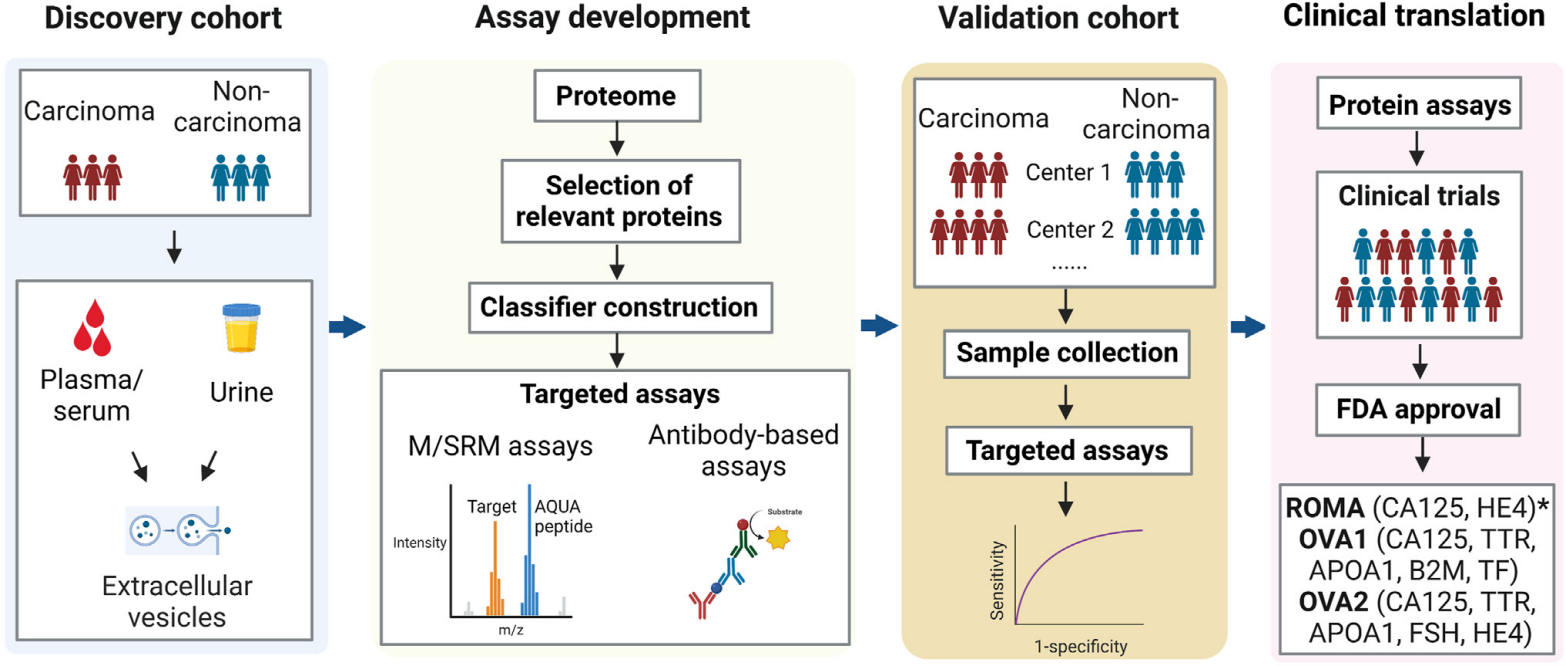
**Schematic workflow for discovering diagnostic biomarkers for ovarian cancer using MS-based proteomics.** ∗, ROMA was approved by FDA, but the biomarkers in RMOA were not first discovered by MS-based proteome. AQUA, Absolute QUAntitation.

We reviewed multiple proteomic studies that tried to identify protein biomarkers for ovarian cancers using liquid specimens, including plasma or serum (77, 78, 79, 80, 81, 82), urine (83, 84, 85, 86), and ascites (87, 88, 89, 90) below (Table 1 and Fig. 1). These specimens are relatively easy to access and minimally invasive, and are therefore widely used in the search for diagnostic biomarkers. However, cancer biomarker concentrations in body fluids, especially in blood, are usually low and technically difficult to identify by MS (91, 92). To achieve comprehensive proteomics profiling of fluid specimens, several strategies can be employed before LC-MS analysis, such as depleting or reducing the concentration of high-abundance proteins using antibodies or nanoparticles (93), and extensive fractionations. Some affinity-based proteomics, such as the targeted immunoassay developed by Olink Proteomics called the proximity extension assay (PEA) (94) and the aptamer-based proteomics assay developed by SomaLogic called SOMAscan Assay (95), are gaining popularity in recent years. In the following section, we review major progress driven by proteomic studies in diagnosing early-stage EOC. Due to length limitations, we regret that not all relevant studies can be cited.

**Table 1:** Diagnostic protein biomarkers of ovarian cancer discovered by MS-based proteomics

Sample type	Biomarker(s)	Discovery				Validation		
		Cohort	Proteomic method	Feature selection	Performance	Cohort	Detection method(s)	Performance
Blood	PPIA^a^ (105)	N (Normal) = 82, N (EOC) = 46	iTRAQ, SAFE-SRM	Empirical-Bayes modified *t* test; Mean squared error by a recursive, leave-one-out cross-validation strategy	Normal: 32/32; Ovarian cancer: 23/28	N (Normal) = 14, N (EOC) = 35	SAFE-SRM	Normal: 14/14; EOC: 20/35
	APOA1, TTR, ITIH4, MUC16 (100)	N (Normal) = 79, N (Borderline) = 13, N (EOC) = 44	SELDI-TOF	Nonlinear unified maximum separability analysis	Training set: 97% specificity, 100% sensitivity; test set: 98% specificity, 86% sensitivity	N (Normal) = 63, N (Benign) = 166, N (EOC) = 138	SELDI-TOF	Specificity (Normal) = 97%, specificity (Benign) = 45%, sensitivity = 78%
	APP^b^, CCL18, CROa, IL-8^b^, and ITIH4^b^ (82)	N (Normal) = 39, N (Benign) = 32, N (EOC) = 41	SELDI-TOF	Decision tree classification	Sensitivity = 97.6, specificity = 91.6	N (Normal) = 190, N (Benign) = 101, N (EOC) = 188	ELISA	Specificity (Normal) = 96.8%, specificity (Benign) = 92.1%, sensitivity = 91.5%
	PROZ (79)	N (Normal) = 54, N (Type I EOC)^c^ = 28, N (Type II EOC)^c^ = 57, N (Borderline)^c^ = 10	iTRAQ, SWATH	Wilcoxon signed rank test	/	N (Normal) = 31, N (Type I EOC)^d^ = 19, N (Type II EOC)^d^ = 30	ELISA	AUC (Type I; CA125+PROZ) = 81%; AUC (Type I; CA125) = 77%; AUC (Type II; CA125+PROZ) = 86%; AUC (Type II; CA125) = 76%;
	LGALS3BP, FGL2, SORT1, TIMP1 and LTBP1 (106)	N (PDX mouse model) = 12; N (Non-engrafted isogenic mice) = 12	N-Glycoproteomics	Human-unique peptides in both PDX-sera and PDX-tumors	/	Two longitudinal serum cohorts (N = 20, n = 96^e^)	PRM	High-degree of correlation between PRM-MS and clinical-ELISA quantitation of CA125 level
	CLIC1, CTSD-30 kDa (77)	Xenograft mouse model of TOV-112D ovarian endometrioid tumor cells (N = 9)	Label-free DDA	Human-specific Proteins Released by Ovarian Tumors	/	N (Normal) = 6, N (Benign) = 9, N (EOC) = 18	MRM; logistic regression analysis	AUC = 0.893
	IGHG2, LGALS3BP, DSG2, L1CAM, THBS1, CA125 (80)	Tissue samples from mice: N (EOC) = 5, N (Normal) = 4; Plasma: N (EOC) = 93; N (Normal) = 80	N-Glycoproteomics; SRM	Consensus logistic regression	AUC = 0.973	Plasma: N (EOC) = 31; N (Normal) = 30	SRM	AUC = 0.99
	MCP-1, IL-8, GROa, CA125 (114)	Ovarian cyst fluid: N (Benign) = 22; N (EOC) = 16	Immunoprecipitation–mass spectrometry	Mann–Whitney U-test	AUC (MCP-1) = 0.82; AUC (IL-8) = 0.80	Paired ovarian cyst fluid and serum: N (EOC) = 78, N (Borderline) = 22, N (Benign) = 156	ELISA; logistic regression analysis	Benign *versus* EOC: AUC (Cyst fluid) = 0.87, AUC (Serum) = 0.88; Benign *versus* borderline: AUC (Cyst fluid) = 0.86, AUC (Serum) = 0.78
	MMP9, IGFBP1 (121)	Ascites: N (EOC) = 10; N (Benign) = 4; serum pools: N (EOC) = 9, N (Normal) = 4, N (Benign) = 5	Label-free DDA	differential expression analysis	/	Serum: N (EOC) = 44, N (Normal) = 78	antibody-based assays	AUC = 0.860
Urine	SPP1, RNASE2 (83)	N (Normal) = 55; N (Benign) = 48; N (EOC) = 42	SELDI-TOF-MS	Student's *t* test	/	N (Normal) = 188; N (Benign) = 52; N (EOC) = 128; N (Other cancer) = 44	ELISA; logistic regression models and “leave-one-out” cross-validation	Specificity = 93%; Sensitivity = 72%
	WFDC2, MSLN, LYPD1, SCGB1A1, IGFBP3, LYVE1, and PTMA (85)	N (EOC) = 10; N (Benign) = 10	Label-free DDA	Differential expression analysis	/	N (EOC) = 10; N (Benign) = 10	PRM	AUC = 0.77–0.95
	WFDC2, PTMA, PVRL4, FIBA, and PVRL2 (86)	N (EOC) = 50; N (benign) = 40	DIA	Random Forest	AUC = 0.970	N (EOC) = 23; N (benign) = 19	DIA	AUC = 0.952
Extracellular vesicles	EPCAM, C1q, ApoE and PLG (119)	Serum: N (EOC) = 10; N (normal) = 10	iTRAQ	Differential expression analysis	AUC for each protein ranged from 0.633 to 0.782	Serum: N (EOC) = 60; N (Normal) = 60	ELISA; Multivariable logistic regression	AUC = 0.913
	GSN, LBP, FGA, and FGG (119)	Plasma: N (EOC) = 3; N (Normal) = 6	TMT	Differential expression analysis	/	Plasma: N (EOC) = 40; N (Normal) = 40	Western blot	AUC (GSN) = 0.8309; AUC (LBP) = 0.6588; AUC (FGA) = 0.8459; AUC (FGG) = 0.7447;
	MYH11, CLCA4, S100A14, S100A2, SERPINB5, IVL, CD109, NNMT, ENPP3 (118)	Uterine liquid biopsy: N (EOC) = 12; N (Normal) = 12	Label-free DDA	Recursive feature elimination (RFE)-Support vector machines (SVM), SVM and ANOVA	Sensitivity = 0.83; Specificity = 1; AUC = 0.99	Uterine liquid biopsy: N (EOC) = 37; N (Normal) = 115	Label-free DDA	Sensitivity = 0.74; Specificity = 0.66; AUC = 0.71

This table includes only those biomarkers validated in independent cohorts.

APOA1, apolipoprotein A1; ApoE, Apolipoprotein E; APP, amyloid beta A4 protein; C1q, Complement C1q; CCL18, chemokine CC2 motif ligand 18; CD109, CD109 antigen; CLCA4, Calcium-activated chloride channel regulator four; CXCL1, CXC chemokine ligand one; CROa, Growth-regulated alpha protein; DSG2, Desmoglein-2; ENPP3, Ectonucleotide pyrophosphatase/phosphodiesterase family member 3; EPCAM, Epithelial cell adhesion molecule; FGA, Fibrinogen alpha chain; FGG, Fibrinogen gamma chain; FGL2, Fibroleukin; GSN, Gelsolin; IGFBP1, Insulin-like growth factor-binding protein one; IGFBP3, Insulin-like growth factor-binding protein three; IGHG2, Immunoglobulin heavy constant gamma two; IL-8, interleukin-8; ITIH4, Inter-alpha-trypsin inhibitor heavy chain H4; IVL, Involucrin; L1CAM, Neural cell adhesion molecule L1; LBP, Lipopolysaccharide-binding protein; LGALS3BP, Galectin-3-binding protein; LTBP1, Latent-transforming growth factor beta-binding protein one; LYPD1, Ly6/PLAUR domain-containing protein one; LYVE1, Lymphatic vessel endothelial hyaluronic acid receptor one; MCP-1, IL-8, Interleukin-8; MMP9, Matrix metalloproteinase-9; MSLN, Mesothelin; MUC16, Mucin-16; MYH11, Myosin-11; NNMT, Nicotinamide N-methyltransferase; PLG, Plasminogen; PPIA, peptidyl-prolyl *cis*-*trans* isomerase A; PROZ, Vitamin K-dependent protein Z; PTMA, Prothymosin alpha; RNASE2, Non-secretory ribonuclease; S100A14, Protein S100-A14; S100A2, Protein S100-A2; SAFE-SRM, sequential analysis of fractionated eluates by SRM; SCGB1A1, Uteroglobin; SERPINB5, Serpin B5; SORT1, Sortilin; SPP1, Osteopontin; THBS1, Thrombospondin-1; TIMP1, Metalloproteinase inhibitor one; TTR, transthyretin; WFDC2, WAP four-disulfide core domain protein two.

a Two peptides from PPIA: VSFELFADK; FEDENFILK.

b These three proteins were included in models by SELDI-TOF but not by ELISA.

c Each with two time points: one at <14 months and the other at >32 months prior to diagnosis.

d Each with longitudinal samples spanning a 7-year period prior to diagnosis.

e Four to five time points for each patient, including diagnosis, post-surgery, post-chemo, remission, and recurrence.

### Serum and Plasma

Serum and plasma are the most common liquid samples used for early diagnosis. Due to their limited specificity and sensitivity, serum CA125 and HE4 individually are not recommended by FDA as early diagnostic biomarkers for EOC but are frequently used to monitor disease progression, evaluate pelvic masses, and estimate the prognosis of patients with EOC (96, 97). Three diagnostic methods based on multiple markers, namely, Risk of Ovarian Malignancy Algorithm (ROMA), OVA1, and OVA2 (trademarked as Overa), have been approved to evaluate the likelihood of malignancy of ovarian adnexal masses (97) (Fig. 1). ROMA integrates serum levels of CA125 and HE4, providing a predictive index that depends on the patient’s menopausal status. ROMA’s sensitivity and specificity for detecting ovarian cancer are 93.8% and 74.9%, respectively (98). OVA1 is an *In Vitro* Diagnostic Multivariate Index Assay using immunoassays of five protein biomarkers, three of which were discovered through proteomic analysis of serum specimens by Daniel Chan’s team (99, 100) (Table 1). Using surface-enhanced laser desorption/ionization (SELDI) analysis coupled with IMAC-Ni ProteinChips, transferrin (TF) in the plasma samples has been reported to provide the best discrimination between patients with ovarian serous neoplasms and those without neoplastic disease by two bioinformatic tools (99). Using SELDI-time-of-flight (SELDI-TOF) MS, three potential biomarkers, apolipoprotein A1 (APOA1), transthyretin (TTR), and inter-α-trypsin inhibitor heavy chain H4 (ITIH4), were found to exhibit consistent dysregulation in the serum specimens of early-stage ovarian cancer samples compared with healthy controls from multiple clinical centers (100). A multivariate predictive model using these three potential biomarkers and CA125 was subsequently established using nonlinear unified maximum separability analysis and further evaluated in an independent validation cohort. This yielded a significantly higher sensitivity (74%) in distinguishing early-stage invasive ovarian cancer from healthy cases compared with models using CA125 alone (65%) at the same fixed specificity (97%) (100). OVA1 incorporates serum levels of five biomarkers, namely CA125, TTR, APOA1, beta two microglobulin (B2M), and TF to generate a probability of malignancy. It may be used by primary care physicians in deciding whether to refer patients with ovarian adnexal masses for specialist evaluation. To improve diagnostic specificity, OVA2 was developed as a second-generation test. In OVA2, the replacement of TF and B2M from OVA1 by follicle-stimulating hormone and human epididymis protein four achieved higher specificity (69% *versus* 54%) and positive predictive value (PPV, 40% *versus* 31%) compared with OVA1 in the same cohort (101). In conjunction with independent clinical and imaging evaluation, OVA2 has been approved as a companion diagnostic test to assess the likelihood of malignancy of ovarian adnexal mass prior to planned surgery.

Another five-peak pattern of uncertain origin fished out by SELDI-TOF MS distinguished ovarian cancer from benign cases with a PPV of 94% in an independent cohort of 116 patients from the same center (102) but has yet to be approved for clinical implementation (103). Overall, these results demonstrate the encouraging utility of proteomics technologies in biomarker discovery for early diagnosis of ovarian cancers. The SELDI-MS has been used about 20 years ago for measuring peptides. However, it is no longer used at the moment, mainly due to the limited sensitivity, reproducibility, and resolution. In addition, SELDI data cannot be used to infer the peptide and protein sequence (104), SELDI-MS is now no longer used in proteomics or peptidomics research as MS-based methods have clearly outperformed methods used 20 years ago in terms of sensitivity, mass accuracy, and resolution. We are optimistic that today's advanced proteomic technologies coupled with careful clinical study design can be expected to generate more robust early diagnostic biomarkers for ovarian cancers.

Targeted proteomics has been used to identify and validate various protein biomarkers for ovarian cancer (80, 90, 105, 106, 107, 108) (Table 1 and Fig. 1). Wang *et al.* (105) developed a sequential analysis of fractionated eluates by SRM, using two-dimensional chromatographic fractionation and parameters optimized for SRM methods based on synthesized peptides of interest. A classifier using two peptides from cyclophilin-A (PPIA) was developed to distinguish ovarian cancer patients from healthy controls or patients with other cancer types (105). This classifier correctly identified all the healthy controls, while 43 out of 63 (68.2%) plasma samples from ovarian cancer patients were correctly identified. To detect low-abundance proteins for HGSOC-leakage biomarker discovery, Sinha *et al.* (106) used a patient-derived xenograft (PDX)-based strategy and N-glycoproteomics to quantify 386 human-unique peptides. These peptides were associated with HGSOC, having been identified in both PDX-sera and PDX-tumors but not in the sera from non-engrafted animals. Using synthetic stable isotope-labeled peptides, a targeted proteomics assay was developed for 408 targeted peptides (including indexed retention time (iRT) peptides and peptides with variable modifications), and used to quantitatively assay two longitudinal serum cohorts of HGSOC patients (106). In both validation cohorts, five peptides (from LGALS3BP, FGL2, SORT1, TIMP1, and LTBP1) mimicked the longitudinal expression of CA125 from diagnosis, surgery, chemo-completion, and remission to recurrence (106). Therefore, this set of five peptides may be a valuable complement to CA125 for monitoring treatment response and cancer recurrence. Importantly, this strategy identified low-abundance biomarker candidates directly secreted by tumors using the entire serum proteome, as human-derived proteins were concentrated in the PDX mouse serum (77, 106). PDX models have the potential advantage of reducing the impact of subject-to-subject heterogeneity at the discovery stage. Recently, Hüttenhain *et al.* (80) explored potential EOC biomarkers using global N-glycoproteomics and a mouse model of endometrioid ovarian cancer. Among their 376 biomarker candidates, 65 proteins with N-glycosites were detectable in human plasma using SRM. After stepwise variable selection and cross-validation using a logistic regression model, five proteins were selected, namely, Ig gamma-2 chain C region, galectin-3-binding protein (LGALS3BP), desmoglein-2, neural cell adhesion molecule L1, and thrombospondin-1. In an independent validation cohort, this 5-protein signature combined with CA125 yielded 94% sensitivity and 93% specificity, while CA125 alone achieved a lower sensitivity (87%) and slightly higher specificity (97%) (80).

### Other Liquid Specimens

Fluids proximal to ovarian cancers, such as ascites and tumor interstitial fluid, have also been analyzed for tumor-secreted proteins as potential biomarkers. Ovarian cancer is the most common primary site for malignant ascites (109). Ascites is caused by impaired fluid drainage and higher net filtration induced by the increased filtration surface and capillary permeability associated with transcoelomic metastases (110). Ascites contains both cellular components from the primary and metastatic tumors (111), and cytokines and chemokines secreted by tumor cells. All these components are potential biomarkers for diagnosis and prognosis (87, 88, 89, 90). Since ascites is only present in advanced EOC, it is not ideal specimen for diagnosis but rather for predicting treatment response.

Ovarian cyst fluid is also a proximate source for biomarker discovery since it likely captures proteins of the initial pathologic processes even before tumor-secreted proteins reach the bloodstream (112). Several studies have profiled the proteome of ovarian tumor fluid to discover candidate biomarkers for diagnosis and prognosis (107, 112, 113, 114).

### Extracellular Vesicles from Liquid Specimens

Exosomes and microvesicles are extracellular vesicles (EVs) released by cells for intercellular communication. They are hypersecreted by tumor cells and tumor microenvironments with resulting effects on altering antitumoral immune responses, angiogenesis, and metastasis formation (115, 116). Thus, they could be valuable biospecimens to discover tumor-specific proteins correlated with tumorigenesis and prognosis. In addition, EVs are isolated from cell culture supernatants (117) and various types of body fluids, such as utero-tubal lavage (118), ascites (88), plasma, and sera (119, 120). Being less invasive to samples than biopsies, EVs are emerging as important clinical specimens for biomarker discovery. Barnabas *et al.* (118) profiled the proteome of microvesicles obtained from liquid uterine biopsies collected before surgery and quantified 8579 proteins. They used these data to build a nine-protein classifier using three algorithms (support vector machine (SVM), recursive feature elimination-SVM, and ANOVA). Among 152 patients in an independent validation cohort, 109 were correctly identified by their classifier with an AUC of 0.71 (118).

Although biomarkers identified in ascites and ovarian cyst fluids cannot be used directly as markers of early-stage cancer, they may guide the prioritization of biomarker candidates identified in the blood for early diagnosis. Nevertheless, multiple studies have reported discrepancies in protein expression in different specimens from the same patients. For example, serum amyloid A-4 (SAA4) and astacin-like metalloendopeptidase (ASTL) discovered in ovarian cyst fluids failed to be validated in plasma by immunoblotting (113). Elschenbroich *et al.* (90) selected 51 candidate protein biomarkers by comparing the proteomes of ascites of serous EOC and benign groups. Four of these proteins, namely, GAPDH, MXRA5, MSLN, and PKM1/2, were precisely quantified using stable isotope dilution-SRM in independent cohorts of ascites and serum samples. GAPDH, MSLN, and PKM1/2 exhibited significant differences in ascites and serum samples. However, smaller changes of protein expression were found in the serum samples compared with ascites (90), which is not surprising since the protein expression in the blood circulation is highly convoluted by proteins released from other tissues. Amon *et al.* (121) found that IGFBP1 was significantly upregulated in the serum and peritoneal fluids of patients with serous ovarian cancer compared with healthy controls and patients with benign ovarian tumors. In an independent set of 122 serum samples, IGFBP1 was shown to correctly classify two groups by ELISA assays with an AUC of 0.860 (121). These results show that ascites and tumor fluids are promising resources of potential biomarkers for the early diagnosis of ovarian cancer.

## Proteomic Characterization of Solid Specimens

Unlike the convoluted protein perturbation data of liquid specimens, proteomes of ovarian tissue specimens obtained from biopsies or surgical excisions provide direct portraits of molecular pathogenesis, including protein patterns associated with tumorigenesis, metastasis formation, and responses to treatment. Such information furthermore provides clues about new therapeutic targets. Molecular pathogenesis of both inter-histotype and intra-histotype tissues coupled with artificial intelligence models aids clinically meaningful patient stratification, thereby promoting precision medicine for different histotypes and molecular subtypes (53, 122). Recently, multi-omics approaches for solid tissue specimens have been increasingly prevalent, enabling biomarker discovery from complementary molecules. More clues regarding the etiopathology and therapeutic targets could be obtained by integrating multi-omics data, such as copy number alterations, *trans*-affected proteins, proteins associated with chromosomal structural abnormalities, and homologous recombination deficiency status (123, 124).

Some studies, especially those focusing on chemoresistance, tried to characterize the molecular alterations based on cell line models (Table 2). Although cell lines are relatively easy to be obtained, expandable, and more homogeneous, however, increasing evidence shows that findings from cell lines may not be directly applicable to patients. In addition, tumor microenvironment cannot be studied using cell lines.

**Table 2:** Dysregulated proteins between resistant and sensitive EOCs discovered by MS-based proteomics

Potential targets	Discovery model	Discovery method	Validation model	Validation method
PKM2, HSPD1 (201)	Cell line: cisplatin-resistant COC1 *versus* parental COC1	iTRAQ	same as discovery	Western blot; RT-PCR; siRNA
ANXA3, DSTN, CFL1, GSTO1, IDH1 (202)	Cell line: sensitive (SKOV3 and A2780) *versus* resistant (SKOV3/CDDP, SKOV3/CBP, A2780/CDDP, and A2780/CBP)	2-DE MALDI-TOF MS	same as discovery	quantitative PCR; western blot
ALCAM, AKAP12, NES (203)	Cell line: sensitive (A2780) *versus* resistant (A2780-CP20)	Label-free DDA	same as discovery	immunoblot analysis
PHB (204)	Cell line: sensitive (SKOV3 and A2780) *versus* resistant (SKOV3/CDDP, SKOV3/CBP, A2780/CDDP, and A2780/CBP)	2-D DIGE; MALDI-TOF-MASS	Cell lines; tissue specimens: N (EOC) = 42	Western blot; immunohistochemistry
TOP1MT (205)	Cell line: doxorubicin sensitive (OVCAR8) *versus* doxorubicin resistant (NCI_ADR/RES)	SILAC	NCI_ADR/RES	RNAi
TXNDC17 (206)	Cell line: sensitive (SKOV3) *versus* resistant (SKOV3-TR30)	Label-free DDA	Tissue specimens: N (EOC) = 157; cell lines	IHC, siRNA, overexpression
PLXDC2 and KRT7 (207)	Tissue specimens: N (paclitaxel-sensitive) = 8; N (paclitaxel-resistant) = 8;	iTRAQ	Tissue specimens: N (paclitaxel-sensitive) = 42; N (paclitaxel-resistant) = 12	Western blot
ANXA3 (202, 208)	Cell line: sensitive (SKOV3 and A2780) *versus* resistant (SKOV3/*Cis*, SKOV3/*Cis*, A2780/Car, and A2780/Car)	2-DE; MALDI-TOF MS	Tissue specimens: N (paclitaxel-sensitive) = 21; N (paclitaxel-resistant) = 21; Cell line: SKOV3	Immunostaining; overexpression
HSP90 (152)	Cell lines: sensitive (TOV-112D, OVSAHO, and MDAH-2774) *versus* resistant (TOV-112D Pt-res, OVSAHO Pt-res, and MDAH-2774 Pt-res)	2D-DIGE; label-free DDA	Cell lines: *ex vivo* in primary cultures derived from Pt-res EOC patients ascites; *in vivo* in a xenograft model	Knock out in cell lines; inhibitor in cell lines, *ex vivo* and *in vivo*
HNRNPA2, GDI2 (209)	Cell line: sensitive (SKOV3) *versus* resistant (SKpac)	2DE; MALDI-TOF; label-free DDA	Cell lines; tissue specimens: N (EOC) = 36, N (benign) = 3	Western blot
NDUFAF2, and YWHAZ (210)	Cell lines: sensitive (SKOV3 and A2780) *versus* resistant (SKOV3-TR and A2780-TR)	LC-FTICR MS	Cell lines. Tissue specimens: N (chemosensitive) = 25; N (chemoresistant) = 21	Cell lines: siRNA; tissue specimens: immunohistochemistry

AKAP12, kinase anchoring protein 12; ALCAM, activated leukocyte cell adhesion molecule; ANXA3, annexin A3; CFL1, cofilin one; DSTN, destrin; GDI2, Rab GDP dissociation inhibitor beta; GSTO1, Glutathione-S-transferase omega 1; HNRNPA2, Heterogeneous nuclear ribonucleoproteins A2; HSP90, heat-shock protein 90; HSPD1, 60 kDa heat shock protein; IDH1, cytosolic NADP+-dependent isocitrate dehydrogenase; KRT7, cytokeratin seven; NDUFAF2, mimitin; NES, nestin; PHB, Prohibitin; PKM2, Pyruvate kinase; PLXDC2, plexin domain containing two; TOP1MT, DNA topoisomerase I; TXNDC17, Thioredoxin domain-containing protein 17; YWHAZ, 14-3-3 protein zeta/delta.

### Differential Diagnosis Driven by MS-Based Proteomics

Most molecular studies have focused on HGSOC, the primary histological type of EOC. However, the other four type I tumors, namely clear cell EOC, endometrioid carcinoma, mucinous carcinoma, and LGSOC, significantly differ from HGSOC in oncogenic mechanisms, precursor lesions, molecular signatures, and optimal treatment approaches (125). Thus, histological type-specific biomarkers for differential diagnosis are essential and instructive for understanding oncogenic mechanisms and developing strategies for precision treatment. Hughes *et al.* (126) optimized a platform for proteomic profiling of FFPE tissues based on a paramagnetic bead technique (Single-Pot Solid-Phase-enhanced Sample Preparation, SP3), and applied this SP3-CTP (clinical tissue proteomics) method for in-depth proteomic analysis of three histological types of ovarian cancer tissues: HGSOC, endometrioid carcinoma, and clear cell EOC. The 500 most variable proteins among these histotypes could segregate them in a PCA plot. On the other hand, the mRNA expression analysis of the same set of genes failed to distinguish HGSOC from endometrioid carcinoma using unsupervised clustering (126), underscoring the importance of proteins for distinguishing phenotypes. Besides known biomarkers of histotypes, cystathionine∼ gamma-lyase (CTH), a candidate protein biomarker highly expressed in clear cell EOC, was validated in cell lines using Western blotting and in three histotypes of ovarian cancer using immunohistochemistry (126). To distinguish mucinous carcinoma from HGSOC at higher sensitivity, Dieters-Castator *et al.* (127) profiled the proteomes of these two histotypes and found four novel biomarker candidates (KIAA1324, PAM, PIGR, and SCGB2A1) with large fold-changes and high specificity, as assessed using the *t* test and a supervised learning model. These four novel biomarkers, coupled with two IHC biomarkers in current clinical use, namely WT and TP53, and two known biomarkers for endometrioid carcinoma, namely PGR and CTNNB1, generated an eight-protein model using nominal logistic regression. The eight-protein model has been validated to outperform both the clinically used and known panels for differential diagnosis of HGSOC and endometrioid carcinoma in an independent cohort (N = 361) (127).

### Adjuvant Diagnosis Driven by MS-Based Proteomics

Molecular classification of cancers stratifies patients into prognostically predefined groups with distinct responses to therapeutic options. Genomic profiling has been reported to help stratify EOC patients by gene mutations and provided clues for promising targeted therapies, such as PARPi for patients with either *BRCA1* or *BRCA2* mutations (128) and MEK inhibitors for patients with LGSOC whose tumors have frequent mutational alterations in the *MAPK* pathway (129, 130, 131, 132). However, HGSOC belongs to C-class cancers, which harbor infrequent mutations but frequent DNA gains and losses. For example, only 23% of HGSOC cases have mutations in *BRCA1/2*, rendering the majority of HGSOC tumors untargetable by the foregoing therapies. Increasing transcriptomic and proteomic subtypes have been reported in EOCs, especially HGSOC (16, 18, 19, 124, 133), with the intent to further stratify these tumors and identify new therapeutic targets or predictive markers.

The Clinical Proteomic Tumor Analysis Consortium has established the most comprehensive proteogenomic data resource so far and has been exploited to propose multiple schemes for HGSOC subtyping. An iTRAQ-based proteomic study by Zhang *et al.* (124) stratified 169 cases of patients with HGSOC into five molecular subtypes in an unsupervised manner, but these subtypes exhibited no significant difference in prognosis. Four of the five subtypes in this study could be matched to independent transcriptome data from TCGA. However, the fifth subtype characterized by extracellular matrix modulations was unique to the proteomic data set, probably because relatively lower tumor purity and secreted proteins could only be detected at the proteomic level (124). Zhang *et al.* then trained a prognosis-associated model using a voting method that combined four parsimonious Cox proportional hazards models based on copy-number alterations of *trans*-affected proteins in 82 of 169 patients. This model consisted of 142 unique proteins which were enriched in proliferation-associated serum response factors and regulation of the actin cytoskeleton (124). This model was further validated in the remaining 87 HGSOC patients, yielding a significant predictive power in OS (*p*-value = 1.9e−6) (124). Hui Zhang’s group further analyzed the N-linked glycoproteome of 119 of the 169 HGSOC patients and stratified them into three molecular subtypes based on glycoproteomic profiles (134). The glycoproteomic stratification correlated with the previous proteome-based stratification scheme and showed differences in prognosis. However, these subtypes remain to be validated in prospective randomized trials.

Besides HGSOC, other uncommon histological types have been investigated. Four molecular subtypes, namely metabolically active, coagulative, fibrotic, and necrotic clusters, were identified in patients with clear cell EOC using proteomics profiling (135). The metabolic subtype was associated with a favorable prognosis, while the coagulative subtype was prognostically poor after correcting for clinical factors (135).

HGSOC is thought to arise from the ovarian surface epithelium (OSE) (136) and the fallopian tube epithelial cells (FTECs) (137, 138, 139, 140), although their proportions remain unclear (141). Some studies (142, 143) have tried to classify the molecular subtypes of HGSOC using proteomics or post-translational modification data of HGSOC putative origin from OSE or FTECs. Coscia *et al.* (142) identified a 67-protein signature that discriminated two molecular subtypes, cluster I (including FTEC isolates) and cluster II (including OSEs), across 30 cell lines (including 26 ovarian cancer, two cervical cancer, two OSE cell lines, and three FTECs) using SVM. This signature was validated in a cohort of HGSOC patients from CPTAC, which segregated into clusters I and II with significantly different OS (142). Compared to cluster II, cluster I cell lines had higher expression of retinoic acid transporter proteins (CRABPs) and enriched vitamin A and retinal binding pathways. The CRABP2-mediated inhibitory effect of all-*trans* retinoic acid on the proliferation of cluster I cell lines was verified *in vitro* and may thus be used to design precision therapy for these subtypes of patients with HGSOC (142). Francavilla *et al.* performed the proteomic and phosphoproteomic profiling of 13 *ex-vivo* primary cells from FTEC, OSE, and HGSOC. They found common alterations of molecules and pathways in FTEC and HGSOC, such as the expression of D-3-phosphoglycerate dehydrogenase PHGDH, activation of CDK7, and enrichment of pathways involved in cell cycle and chromosome organization. However, they found little overlap in dysregulated proteins and pathways between OSE and HGSOC, suggesting FTECs may be the primary cell type of origin of HGSOC (143). CDK7 is a potential target for intervention since both its inhibitor (THZ1) and siRNA reduced the proliferation of HGSOC cell lines (143). Thus, HGSOC subtyping based on their cell type of origin also aids patient stratification and provides leads for new therapeutic targets.

## Therapeutic Targets Discovered by MS-Based Proteomics

Proteomics has proven to be an effective tool to discover potential therapeutic targets for ovarian cancers. Ovarian cancer tissues are the primary specimens for identifying druggable candidates. In the following, we summarize some of the most relevant draggable target candidates discovered by MS-based proteomics.

Phosphoproteomic data of 83 HGSOC and 20 normal fallopian tube samples found dysregulated activation of proliferation-associated cyclin-dependent kinases (CDKs) and mitotic kinase aurora kinase A (AURKA) (144). EntreMed Inc developed a small molecule compound, ENMD-2076, which selectively inhibits AURKA and several kinases involved in angiogenesis, including VEGFRs and FGFRs. Two phase 2 clinical trials have evaluated its activity in recurrent, platinum-resistant EOCs (145) and clear cell EOCs (146). Preliminary antitumor activity of daily oral ENMD-2076 has been reported in chemoresistant EOCs with 22% of a 6-months PFS rate (145), while single-agent ENMD-2076 had limited efficacy against clear cell EOCs (146). Clinical trials enrolling larger cohorts are needed to evaluate the efficacy of ENMD-2076 as well as its synergistic effect with chemotherapies.

Multiple diagnostic markers discovered from biofluid samples, including MSLN in urine (85) and EPCAM in extracellular vesicles from serum (119) (Table 1), have been selected as targets for antibody-based therapies to reduce systemic toxicity based on their overexpression in EOC tissues. The trifunctional antibody catumaxomab (trademarked Removab) has been approved by the European Medicines Agency for intraperitoneal treatment of malignant ascites due to EPCAM-positive cancers when standard therapy is unavailable. Ovarian cancer is an EPCAM-positive cancer and some clinical trials have reported symptomatic improvement in patients with advanced EOC having malignant ascites (147, 148). In addition, multiple MSLN-directed antibody-drug conjugates (ADCs) have been developed, and some clinical trials have shown good tolerance and preliminary evidence of antitumor activity in ovarian cancer (149, 150, 151).

Multiple differentially dysregulated proteins have been found between resistant and sensitive ovarian cancer cell lines or patient tissue samples (Table 2). Heat-shock protein 90 (HSP90) was identified to be upregulated in platinum-resistant EOC cell lines compared with their parental cells (152). HSP90 ablation or inhibition resensitized platinum-resistant cells to cisplatin (152). In addition, *in vivo* synergistic antitumor effects of cisplatin and HSP90 inhibitor were observed in mice bearing xenograft tumors (152). However, a phase 2 clinical trial of gemcitabine in combination with tanespimycin, an HSP90 inhibitor (ClinicalTrials.gov Identifier: NCT00093496), showed only limited antitumor activity in advanced EOCs (153).

Coscia *et al.* (154) compared proteome of FFPE tumor samples from chemoresistant and chemosensitive HGSOC patients, and identified cancer/testis antigen 45 (CT45) as a favorable prognosis biomarker. This study proposed CT45 as a potential therapeutic target on the basis of its synergistic effect on carboplatin-induced hyperactivation of DNA damage pathways, and its role as a cancer antigen to facilitate anti-tumor cytotoxic T cells (154).

Multiple cancer-testis antigens (CTAs), including CT45, NY-ESO-1, and MAGE-A, are physiologically expressed only in immunologically privileged testicular tissue. They are also expressed in multiple types of carcinomas and show good immunogenicity and thus could be used to develop tumor vaccines (155). Two clinical trials (ClinicalTrials.gov Identifiers: NCT00066729, NCT00616941) have detected vaccine-induced T cells and specific antibody responses against NY-ESO-1-expressing tumor cells in EOC patients vaccinated with the NY-ESO-1 peptide epitope (156, 157) and recombinant poxviruses expressing NY-ESO-1 antigen (158). In addition, decitabine, a DNA methyltransferase inhibitor (DNMTi), further enhances these immune responses in patients treated with NY-ESO-1 vaccine and chemotherapy of phase 1 clinical trial (ClinicalTrials.gov Identifier: NCT00887796), which may be due to the upregulation of CTAs by DNMTi (159). A retrospective analysis of these clinical trials found improved overall survival in NY-ESO-1-positive patients when treated with NY-EOS-1-specific vaccines (160). Multiple clinical trials using immunotherapeutic approaches based on NY-ESO-1, including vaccines and adoptive T-cell therapies, are ongoing in ovarian cancer patients (161).

Eckert *et al.* (162) performed proteomic profiling of tumor and stromal compartments microdissected from FFPE HGSOC specimens taken from four anatomic sites. Methyltransferase nicotinamide N-methyltransferase (NNMT) was significantly upregulated in the stromal compartments of omental metastases compared with primary stromal sites (invasive fallopian tube or ovarian lesion). Knockdown of NNMF in cancer-associated fibroblasts reversed the cancer-associated fibroblast phenotype, inhibited cancer cell proliferation *in vitro*, and reduced the tumor burden *in vivo* (162). NNMF inhibitor, 5-amino-1-methylquinolin-1-ium, exhibited efficacy in relieving tumor burden in a mouse model of ovarian cancer metastasis (162). No clinical trial targeting NNMT is available yet.

## Clinical Trials for Ovarian Cancer

**Fig. 2:**
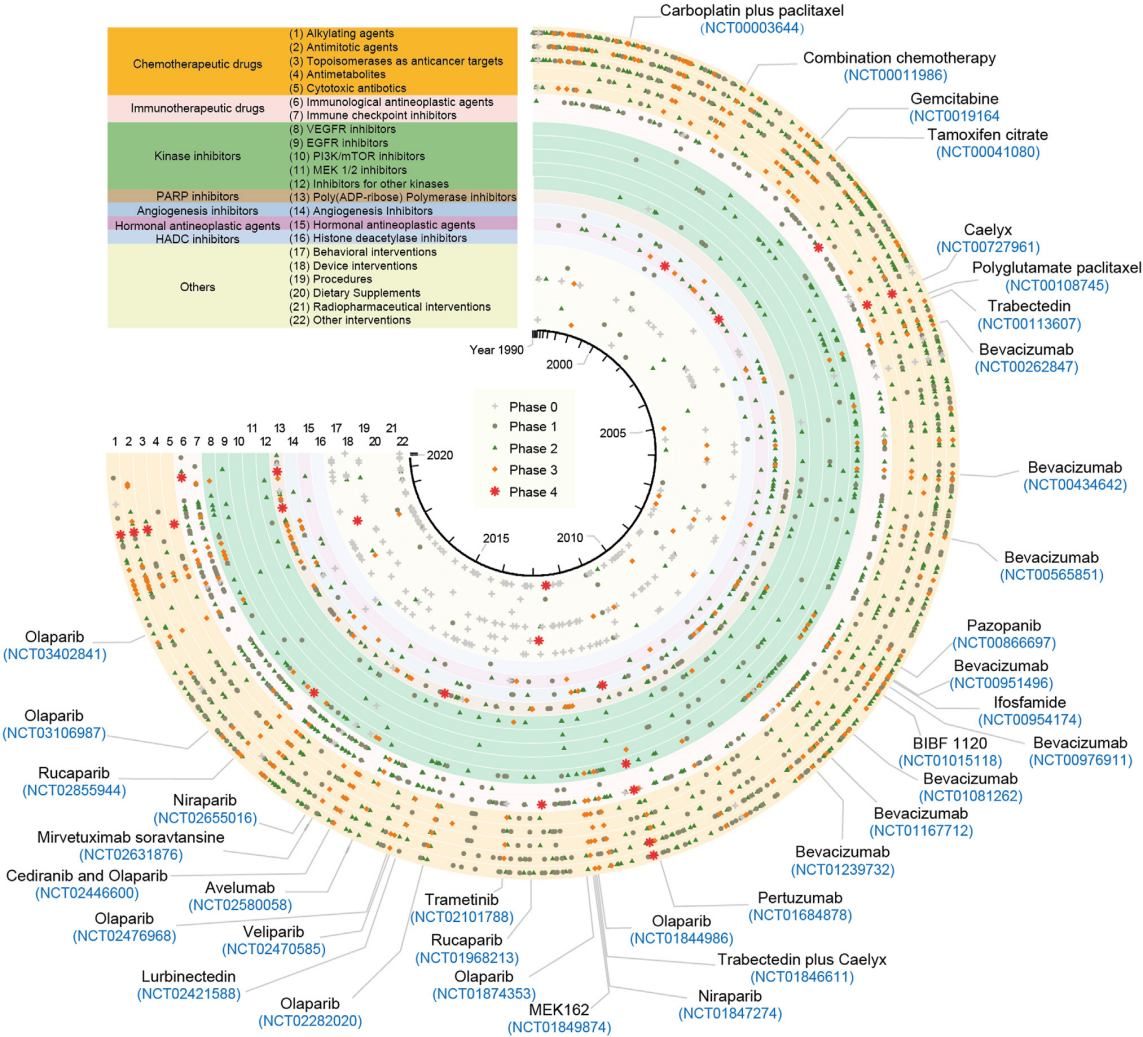
**Summary of 1418 interventional clinical trials of ovarian cancer which have been completed or at active but not recruiting status according to the****ClinicalTrials.gov****database.** The clinical trials are ranked clockwise by the start year, and labeled according to their phases and types of interventions. Phase 0 represents early phase 1 or those without applicable phases as defined by FDA. The clinical trial identifiers and studied drugs of 37 clinical trials in phase 3 and phase 4 which investigated anti-neoplasm medications and have posted detailed results in the database were labeled.

The ClinicalTrials.gov database shows over 2500 clinical trials in ovarian cancers conducted globally since 1990. Among these, 1418 interventional studies have been completed or are still active but not recruiting more patients (Fig. 2 and supplemental Table S1). 348 trials have submitted detailed results to the database. The number of new clinical trials increased annually and peaked in 2017 but declined significantly in the past 2 years, probably because of the COVID-19 pandemic. After excluding the clinical trials without applicable phases as defined by FDA, around 35% are in phase 1, which focuses on the safety of the interventions; 52% reached phase 2 which evaluates both effectiveness and safety. Only 12.06% are in phase 3 and 1.38% in phase 4. 37 clinical trials in phases 3 and 4 investigated anti-neoplastic medications for ovarian cancer and have posted their results (Fig. 2 and supplemental Table S1). Among these 37 clinical trials, 18 focused on resistant or relapsing patients, or on maintenance therapy to prevent a recurrence. Twelve of 37 trials focused on PARP, 10 on VEGFR, nine studied chemotherapy, one modulated sex hormone levels, and the remaining five investigated other molecules, including the MEK inhibitor, the anti-PD-L1 antibody, the ERBB family inhibitor, and an ADC targeted against FRα. Pertuzumab is a monoclonal antibody against the extracellular domain of the human epidermal growth factor receptor 2 (HER2) and inhibits its heterodimerization with other HER receptors, especially HER3. Despite promising phase 2 data of Pertuzumab in platinum-resistant EOC patients with low HER3 mRNA expression (163), this treatment did not improve overall survival in the phase 3 trial, PENELOPE (ClinicalTrials.gov Identifier: NCT01684878) (164, 165). Both trametinib and binimetinib are selective inhibitors of MEK1/2. Trametinib improved PFS in patients with recurrent LGSOC in phase 2 and 3 trials (ClinicalTrials.gov Identifier: NCT02101788), while no statistically significant relationship was observed between *MAPK* pathway aberrations and prognosis (166). However, the MILO/ENGOT-ov11 study of binimetinib in recurrent or persistent LGSOC did not meet its PFS endpoint compared to physicians’ choice chemotherapy (131). JAVELIN Ovarian 200 evaluated the efficacy of Avelumab, the anti-PD-L1 antibody, alone and its combination with chemotherapy in patients with platinum-resistant or -refractory ovarian cancer. This study, however, reported no significant improved outcomes (39). Mirvetuximab soravtansine (MIRV) is an ADC comprising an FRα-binding antibody and the tubulin-targeting agent Ravtansine. No statistically significant improvements in PFS were detected in platinum-resistant EOC patients who received MIRV compared with chemotherapy. However, all the secondary endpoints of the MIRV group outperformed those with chemotherapy, especially in patients with high FRα expression (167). Thus, except for MEKi, no new targeted drug has achieved improvements in PFS or OS when compared with treatments approved by FDA. Notably, we found that although these clinical trials for targeted therapies have tried to select patients for the various arms of the study based on the presence of specific mutations (*i.e.*, *MAPK* pathway aberrations for trametinib) or dysregulated gene expression (*i.e.*, mRNA expression of HER family for Pertuzumab, PD-L1 expression for Avelumab, and FRα expression for MIRV), most of them gained little benefits from a preselection of molecular subtypes, indicating the presence of multiple confounding factors. More comprehensive multi-omics approaches including proteomics are required to understand the protein network and develop computational models to better predict the drug responsiveness (168).

Among the 1418 interventional studies, chemotherapies are the main interventions (48.30%, including 406 with alkylating agents, 134 with antimetabolites, 375 with antimitotic agents, 208 with topoisomerase inhibitors, and 121 with cytotoxic antibiotics), followed by immunological agents (23.13%, including 90 with immune checkpoint inhibitors), kinase inhibitors (17.98%, including 153 with VEGFR inhibitors, 35 with ERBB family inhibitors, 24 with PI3K/mTOR inhibitors, and three with MEK inhibitors), PARPi (8.39%), angiogenesis inhibitors (7.76%), hormonal therapies (4.30%), and 11 studies targeting histone deacetylases (Fig. 2 and supplemental Table S1). Some targets, such as CLDN6 (169), EZH2 (170), mesothelin (171), TRPV6 (172), GDF15 (173), SHP2 (174), DLL3 (175), and alpha-folate receptor (176, 177) were overexpressed in ovarian cancer or associated with unfavorable prognosis. Multiple small molecules or ADCs have been developed to suppress these proteins in clinical trials. However, none of these targets was first discovered by proteomic profiling, and none of their early phase clinical trials progressed to phases 3 and 4. Considering the large number of dysregulated proteins identified by MS-based proteomics, we expect a good proportion may advance to clinical trials as novel therapeutic targets supported by solid preclinical validations in disease models.

## Outlook

Proteins are robust biomarkers for disease diagnosis and prognosis and are the direct targets for most drugs. Biofluid specimens are frequently used for disease screening. Ultra-high-speed proteomic profiling enabling analysis of a blood proteome within a few minutes has been proposed. However, most low-abundance proteins are not measured with this approach (178). Deeper proteomic profiling with TMTpro or nanoparticles allows measurement of over 1000 circulating proteins but still takes several hours to analyze each specimen (93, 179, 180). Recently, non-mass spectrometry methods, such as PEA (94) and SOMAscan Assay (181), are gaining popularity in analyzing liquid specimens due to their increased proteomic depth and throughput. These methods could be potentially used to identify biomarkers for early detection and recurrence of EOCs (182, 183, 184, 185, 186, 187).

Urine is a more accessible biofluid specimen from which more proteins may be identified. The urine proteome covers 80% of serum proteins and contains valuable physiological and pathological information (188). Thus, urine could be a choice biospecimen to explore early diagnostic biomarkers for EOCs. However, urine has relatively variable protein concentration which may fluctuate depending on the physiological and pathological status of an individual. More efforts are required to build up community consensus for urine-based biomarkers (189).

From bulk tissue specimens, many potential biomarkers and therapeutic targets have been identified by MS-based proteomics (Tables 1 and 2). However, few of them have been advanced to clinical applications, partly because the experimental design of most studies does not meet the rigorous requirements of clinical trials. In addition, most studies do not have a sufficient sample size. For example, robust measurement of proteins in small amounts of clinical specimens is required, and DIA-MS is increasingly used for protein biomarker discovery due to its superior sensitivity, reproducibility, and robustness (53, 57, 190). Thus, we anticipate that application of DIA-MS in analyzing carefully curated clinical cohorts will greatly enhance the discovery of ovarian cancer biomarkers.

Despite the existence of multiple schemes of unsupervised molecular subtyping for patients with HGSOC based on various types of ‘omics data profiles, none has been translated into clinical practice, partly because it is difficult to unambiguously assign a single HGSOC tumor to a single subtype due to subclonal heterogeneity (20). For example, the expansion and evolution of pre-existing drug-tolerant subclones could lead to intra-tumoral heterogeneity and acquired resistance (191). In addition, the tumor microenvironment also influences the efficacy of immunotherapy. Disappointing results of clinical trials of checkpoint inhibitors in patients with advanced-stage ovarian cancer (ClinicalTrials.gov Identifiers: NCT02580058, NCT03038100) (39, 40) could probably be ascribed to the complexity of tumor microenvironments.

Spatial dissection and molecular characterization of dominant tumorous subclones and the microenvironment are crucial to therapy selection, and therefore essential for the precision stratification of these tumors. We anticipate emerging spatial and single-cell ‘omics technologies, in particular proteomics (59, 61, 192, 193), will likely greatly advance this field in the near future. Spatial proteomics not only helps to discover biomarkers that are rendered elusive by tissue homogenization, but could potentially also identify secreted proteins in the ECM which are not usually detected in transcriptome experiments. Multiple strategies for spatial proteomics have been developed recently, including laser capture microdissection-based methods (194), tissue expansion-based methods (61, 195), and the micro-scaffold-assisted method (196). Recently, overexpression of NNMF, a potential therapeutic target, has been reported only in the stromal compartment but not in tumor cells, underscoring the importance of spatial proteomics (162). Other single-cell “omics technologies” have been applied in EOC. For instance, a single-cell landscape of HGSOC from the ascites ecosystem has been characterized using single-cell RNA seq, uncovering inter- and intra-patient patterns of heterogeneity (197). However, transcriptomes and proteomes correlate poorly as a result of multiple modifying processes including post-transcriptional processing, post-translational modification, and protein degradation (198). Single-cell proteomics might offer more valuable information (59). Gonzalez *et al.* (199) used single-cell mass cytometry to gain insight into the heterogeneity of HGSOC specimens and found that a cellular subset co-expressing vimentin with high levels of HE4 and cMyc contributed to carboplatin resistance. Their single-cell analysis covered only 41 proteins. With rapid advances in MS-based single-cell proteomics, one to two thousand proteins have been quantified (59, 62). Although the throughput of these emerging MS-based single-cell proteomics technologies is still limited, it exhibited higher stability in quantification when compared to those of single-cell RNA-seq (59), which allows us to draw biological conclusions from much fewer cells (200). Therefore, the uncertainties in EOC stratification and treatment can be expected to be further clarified at the single-cell level. The dominant subclones could be potentially targeted by drugs selected based on their dysregulated oncogenic signaling pathways which could be inferred from single-cell and/or spatial proteomic data. In the meantime, we may also identify the subclone with a worse prognosis, and design customized therapies to minimize drug resistance and metastasis. In addition, spatial proteomics can also shed light on immune responses from the tumor microenvironment, which could potentially guide immunotherapies against the tumor. Thus, despite throughput limitations, single-cell proteomics still provided complementary information to single-cell RNA-seq.

## Supplemental Data

This article contains supplemental data.

## Conflict of interest

The authors declare the following financial interests/personal relationships which may be considered as potential competing interests: T. G. is the founder of Westlake Omics Inc. The other authors declare no competing interests.
